# Prediction of Patient Survival in Cases of Acute Paraquat Poisoning

**DOI:** 10.1371/journal.pone.0111674

**Published:** 2014-11-21

**Authors:** Sae-Yong Hong, Ji-Sung Lee, In O. Sun, Kwang-Young Lee, Hyo-Wook Gil

**Affiliations:** 1 Department of Internal Medicine, Soonchunhyang University Cheonan Hospital, Cheonan, Republic of Korea; 2 Biostatistical Consulting Unit, Soonchunhyang University Medical Center, Seoul, Korea; 3 Department of Internal Medicine, Presbyterian Medical Center, Jeonju, Republic of Korea; Carleton University, Canada

## Abstract

Paraquat concentration-time data have been used to predict the clinical outcome following ingestion. However, these studies have included only small populations, although paraquat poisoning has a very high mortality rate. The purpose of this study was to develop a simple and reliable model to predict survival according to the time interval post-ingestion in patients with acute paraquat poisoning. Data were retrospectively collected for patients who were admitted with paraquat poisoning to Soonchunhyang University Choenan Hospital between January 2005 and December 2012. Plasma paraquat levels were measured using high-performance liquid chromatography. To validate the model we developed, we used external data from 788 subjects admitted to the Presbyterian Medical Center, Jeonju, Korea, between January 2007 and December 2012. Two thousand one hundred thirty six patients were included in this study. The overall survival rate was 44% (939/2136). The probability of survival for any specified time and concentration could be predicted as (exp(*logit*))/(1+exp(*logit*)), where *logit* = 1.3544+[−3.4688×*log10*(*plasma paraquat* μg/M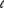)]+[−2.3169×*log*10(*hours since ingestion*)]. The external validation study showed that our model was highly accurate for the prediction of survival (C statics 0.964; 95% CI [0.952–0.975]). We have developed a model that is effective for predicting survival after paraquat intoxication.

## Introduction

Paraquat (1,1′-dimethyl-4,4′-bipyridium dichloride; PQ) is a non-selective herbicide. The fatality rate of PQ intoxication remains high due to the lack of an effective treatment. It has been proposed that plasma PQ level might be a reliable predictor of prognosis [1]–[3]. Proudfoot examined only 79 cases of PQ poisoning and related outcomes to plasma PQ concentration on admission and the ingestion to sampling interval [2]. Hart proposed a nomogram to predict outcome, on the basis of data from 218 patients with PQ poisoning [3]. This curve has been widely used in clinical situations. More recently, some studies have suggested some predictive equations based on similar sample sizes [4]–[9]. However, these equations have not been widely used in clinical situations because they are hard to calculate. The number of patients in investigations on this topic should be large enough to ensure statistical significance in the results. We considered that previous data require verification in a sufficiently large population. The plasma level of PQ peaks early, usually within 2–4 h after ingestion, followed by a rapid decline with a steep gradient due to rapid distribution of PQ from the circulation to other compartments [10]. In the clinical situation, plasma PQ level within 12 h may be reliable but after that, the curve in the nomogram is not discriminable, so it becomes hard to predict the clinical outcome. Therefore, there is a need for a new and simple validated nomogram to predict mortality.

The purpose of this study was to develop a simple and reliable model to predict prognosis according to plasma PQ concentration and time interval in patients with acute PQ poisoning.

## Materials and Methods

The study subjects were patients with acute PQ poisoning who were admitted to the Institute of Pesticide Poisoning of Soonchunhyang University Cheonan Hospital, Cheonan, Korea, from January 2005 to December 2012. Soonchunhyang Cheonan Hospital's Investigational Review Board approved this study.

Plasma PQ levels were measured by high-performance liquid chromatography. The treatment protocol was previously reported [11]–[16]. In brief, the treatment's principle was (1) extracorporeal elimination, (2) intravenous antioxidant administration, (3) diuresis with a fluid, and (4) cytotoxic drugs. We developed three models to predict mortality according to the interval after ingestion. Model 1 was based on the plasma PQ concentration and survival data of patients who were admitted to our hospital within 12 h after ingestion of PQ. Model 2 based on the plasma PQ concentration and survival data of patients who were admitted to our hospital within 24 h after ingestion of PQ. Model 3 was based on the plasma PQ concentration and survival data of all patients who were admitted to our hospital at any time after ingestion of PQ. The parameters were the plasma PQ level on admission and interval after ingestion. The outcome was death or survival. Survival was defined as previously reported [11].

Multivariable logistic regression analysis was used to develop a prediction model for survival outcome. Internal validation was performed using bootstrapping, and external validation of the model was performed using data from patients admitted between January 2007 and December 2012, which had been collected at the Presbyterian Medical Center, Jeonju, Korea.

Analyses were performed using R-project 2.11.1 (package ‘rms’ version 4.11). Discrimination statistics such as C-statistics (equivalent to the area under the receiver operating characteristic curve) were provided to indicate how well an entire model matched with the observed values. Calibration of the model was assessed by the Hosmer-Lemeshow test and by the plots comparing predicted versus observed probability of outcome.

### Ethics statement

Soonchunhyang Cheonan Hospital's Investigational Review Board approved this study. Informed consent was waived by the board.

## Results

Two thousand three hundred fifty six patients presented between January 2005 to December 2012, but only 2136 patients were included in this study. The remaining 220 patients were excluded because they were transferred to another hospital or their initial PQ level was not available.

The mean age was 51.29±16.37 yr. The mean PQ level was 26.67±69.46 µg/ml (range, 0.01–925.91 µg/ml). The mean time from ingestion was 17.24±30.79 h (range, 0.2–360 h). A total of 1556 patients were admitted ≤12 h after ingestion of PQ and 199 were admitted between 12 and 24 h after PQ ingestion. Three hundred and eighty-one patients were admitted more than 24 h after PQ ingestion. The overall survival rate was 44% (939/2136).

To calculate the predicted probability of survival for any specified time and concentration, a formula was derived based on the logistic regression coefficients (Table 1). Our logistic regression of log-transformed data yielded the following probability formula:

**Table 1:** Prediction model with paraquat concentrations and time interval.

		Model 1	Model 2	Model 3
Overall performance	R^2^	0.846	0.833	0.811
	Brier	0.050	0.056	0.065
	Brier_scaled_	0.784	0.763	0.735
Discrimination	c statistic	0.981	0.977	0.970
	[95% CI]	[0.975–0.986]	[0.972–0.983]	[0.964–0.976]

Model 1: patients who were admitted to our hospital within 12 h after ingestion of paraquat.

Model 2: patients who were admitted to our hospital within 24 h after ingestion of paraquat.

Model 3: all patients who were admitted our hospital at any time after ingestion of paraquat.

Probability of survival = (exp*(logit)*)/(1+exp*(logit)*).

The developed models were as follows:

Model 1. 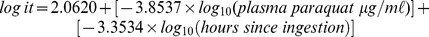


Model 2. 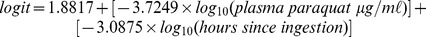


Model 3. 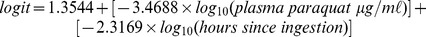


**Figure 1:**
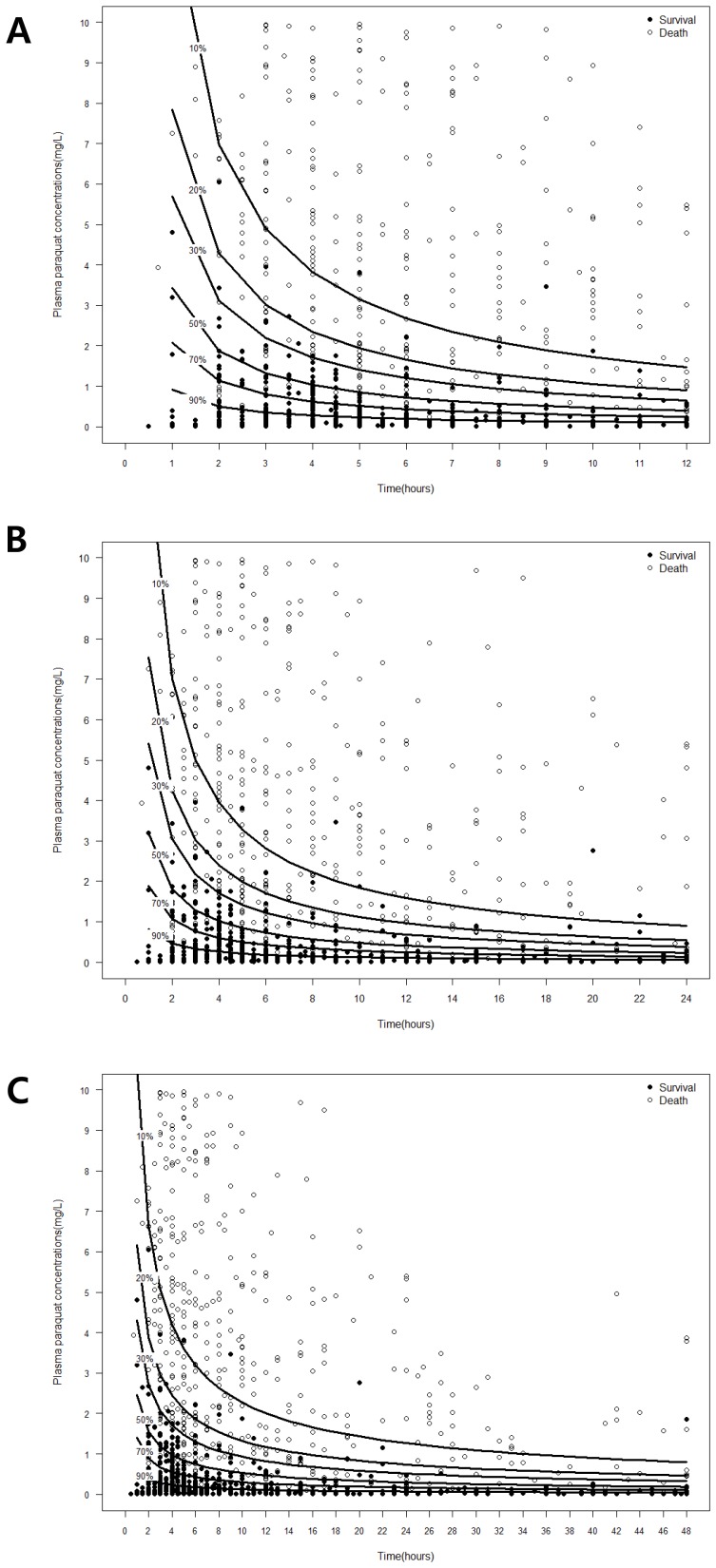
Contour graph showing relation between plasma paraquat concentration (µg/ml), time after ingestion, and probability of survival. A. Probability curve of patients who were admitted to our hospital within 12 h after ingestion of paraquat. B. Probability curve of patients who were admitted to our hospital within 24 h after ingestion of paraquat. C. Probability curve of all patients who were admitted to our hospital at any time after ingestion of paraquat.

The nomogram shown in Figure 1 is based on our three models. The probability of survival is easily determined from the nomogram according to time and concentration.

Table 2 shows the results of internal and external validation. All three models provided accurate survival predictions. Of the three models, Model 1 is statistically the most accurate. These data show that the nomogram of model 1 (Figure 1A) is the most validated survival curve and discriminating curve.

**Table 2:** Internal and external validation of our developed model.

Internal validation		Model 1	Model 2	Model 3
Calibration	Calibration Intercept	0.0003	0.0028	0.0004
	Calibration slope	0.9959	0.9981	0.9931
**External validation**				
Discrimination	c statistic	0.965	0.964	0.964
	[95% CI]	[0.953–0.977]	[0.952–0.976]	[0.952–0.975]

Model 1: patients who were admitted to our hospital within 12 h after ingestion of paraquat.

Model 2: patients who were admitted to our hospital within 24 h after ingestion of paraquat.

Model 3: all patients who were admitted to our hospital at any time after ingestion of paraquat.

## Discussion

The purpose of our study was to develop a reliable model to predict survival after PQ poisoning. A reliable predictor of prognosis would be helpful to guide treatment and investigate the efficacy of new treatments. Our center reported initial laboratory parameters related to the prognosis of patients with acute PQ poisoning [12]. Plasma PQ concentration and creatinine level at admission are important prognostic markers [13]. Some oxidative stress markers have been investigated by our group and in other reports [14]–[19], but it is doubtful that these markers would be superior to plasma PQ concentration. In addition, although these markers may be involved in the pathogenesis in PQ injury, they are not useful in the clinical situation because they require expensive laboratory capabilities. Thus, PQ concentration–time data have been used to predict outcome for nearly three decades. Overall, the plasma PQ concentration seems likely to remain the most useful marker of exposure and severity.

Some prediction models based on plasma PQ concentration have been suggested previously. Among these, Proudfoot and Hart, who are pioneers in this field, reported nomograms that are simple to apply in the clinical situation [2],[3], but their prediction models were based on very small sample sizes. A large sample size is required to develop a model for predicting survival in PQ poisoning, because of the high mortality associated with this poisoning.

We developed three models in this study. The models, especially model 1, were very effective for prediction of survival after PQ exposure. Nomogram of model 3 showed that the curve became an asymptote after 24 h, so we consider that models 1and 2 might be more useful in clinical situations. Compared with previous model, our nomogram showed well discriminate curve. Our data suggest that physicians could select one model according to the time interval between PQ ingestion and admission to hospital. Our nomogram is simple to use, and likely to be useful in clinical situations.

For the purpose of external validation, we used other Korean data from 788 patients with acute PQ poisoning. Our models yielded accurate results in this large population. Our prediction model could be used as a research tool to compare new treatments and previous modalities.

Our study has some limitations. This is a retrospective study and the study population comprised only Asian people. The treatment protocol was changed during the period over which the data were collected. Extracorporeal therapy and antioxidant agents were applied to patients with PQ poisoning over the whole period, but immune suppression pulse therapy including cyclophosphamide was not applied prior to 2009. It was not our intent to focus on comparison between treatments. Our focus was to develop a new prediction model.

In conclusion, we consider that our equation and nomogram are reliable and accurate for the purpose of predicting survival in patients with acute PQ poisoning. This model can be used in clinical situations and as a research tool for studies on the efficacy of new treatment.
